# Crystal structure of potassium chloride monohydrate: water intercalation into the B1 structure of KCl under high pressure

**DOI:** 10.1107/S2053229622011135

**Published:** 2022-12-01

**Authors:** Keishiro Yamashita, Kazuki Komatsu, Hiroyuki Kagi

**Affiliations:** a Geochemical Research Center, Graduate School of Science, The University of Tokyo, Hongo 7-3-1, Bunkyo-ku, Tokyo 113-0033, Japan; b Institute of Physical Chemistry, University of Innsbruck, Innrain 52c, Innsbruck, 6020, Austria; University of the Witwatersrand, South Africa

**Keywords:** salt hydrate, high pressure, intercalation, crystal structure, potassium chloride

## Abstract

A new hydrate form of potassium chloride, KCl·H_2_O, is identified for the first time by *in situ* single-crystal X-ray diffraction under high pressure. It has a monoclinic structure with lattice parameters of *a* = 5.687 (7), *b* = 6.3969 (3), *c* = 8.447 (3) Å and β = 107.08 (8)° at 2.23 (4) GPa and 295 K. The structure of this hydrate has K—Cl alignments similar to the B1 phase of anhydrous KCl, while water molecules intercalate among the ionic species. The coordination structures of the K and Cl atoms can be regarded as the intermediate states between the B1 and B2 phases of KCl. This finding provides a perspective on the structural interpretation of multicomponent materials and an additional candidate for bound water in salt–water systems under high pressure, such as inside of icy bodies.

## Abstract 2

A new potassium chloride monohydrate, KCl·H_2_O, is discovered under pressure. The crystal structure is determined by *in situ* single-crystal X-ray diffraction and its structure can be explained as the water-incorporated B1-type anhydrous KCl.

## Introduction

Potassium chloride (KCl) does not form any hydrate at ambient pressure. Other alkali halides have limited numbers of hydrates as well, such as the case of sodium chloride, which only forms the dihydrate at low tem­per­atures (Klewe & Pedersen, 1974 ▸). These are in contrast to some other salts, such as LiCl and MgCl_2_, which form various hydrates (*e.g.* Sohr *et al.*, 2018 ▸; Hennings *et al.*, 2013 ▸). The low hydrate-forming capability of KCl can be ascribed to the energetic disadvantages of hydration, as seen in its negative enthalpy of dissolution. The dissolution of KCl in water is entropy driven, whereas other salts, like MgCl_2_, have an enthalpy gain for dis­solution. Hence, KCl prefers the anhydrous form rather than forming a hydrate.

Anhydrous KCl adopts different crystal structures under different circumstances. At ambient conditions, anhydrous KCl has a face-centred-cubic (f.c.c.) structure with *Fm* 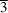 *m* sym­metry called the B1 phase. KCl transforms into the B2 phase with a simple-cubic structure and *Pm* 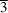 *m* symmetry at around 2–3 GPa, reducing the volume by ∼12% (Vaidya & Kennedy, 1971 ▸; Campbell & Heinz, 1991 ▸). In the B1→B2 transition, the coordination number increases from six to eight with the increase in K—Cl distances. The structural change of simple ionic crystals can be simply explained by the *r*_C_/*r*_A_ ratio (*r*_C_ and *r*_A_ are the ionic radii of the cations and anions, respectively). Because of the larger compressibility of the anions with respect to the cations, the B2 phase is favoured under high pressure for a higher *r*_C_/*r*_A_ ratio than under ambient conditions. On the other hand, the structure of hydrates is more complicated than that of anhydrous salts, con­sisting of oxygen–cation coordination, hydro­gen bonds and covalent bonds related to the water molecules, rather than a single type of interaction like ionic bonding. The differences among the interactions in the hydrate crystals result in anisotropic responses to stimulations, such as thermal expansion and compressibility. The physical properties of the overall crystal reflect the packing scheme of the atomic species (*e.g.* Fortes *et al.*, 2017*a* ▸). Such structure-dependent properties play an essential role in various materials but are difficult to predict from scratch. Experimental elucidations are demanded with compensation by computational evaluation, but such structural studies of salt hydrates under high pressure are still limited.

We report here a new hydrate of potassium chloride which is stable only under high pressure. This phase was discovered unexpectedly from a concentrated KCl solution under high pressure at ambient tem­per­ature. Its structure was determined by the combination of X-ray single-crystal diffraction and density functional theory (DFT) calculations.

## Experimental

### Single-crystal X-ray diffraction under high pressure

A saturated KCl solution, corresponding to 26.5 wt% at 298 K and atmospheric pressure (Pinho & Macedo, 2005 ▸), was prepared by dissolving an excess amount of reagent-grade KCl (99.5%) purchased from Wako Corporation in Milli-Q water. The solution was loaded into a diamond anvil cell (DAC) with a small amount of crystalline KCl to achieve the desired measurement conditions, whose details are described later. A pair of Boehler–Almax-type diamond anvils (Boehler & De Hantsetters, 2004 ▸) with a culet diameter of 600 µm were used. Stainless steel (SUS301) plates were used as a gasket with a ϕ = 400 µm hole as a sample space. To obtain high-quality X-ray diffraction data, a PFA (Teflon PFA) ring with an inner diameter of 200 µm was introduced as an inner gasket and a modified ‘clover seat’ backing seat was used (Komatsu *et al.*, 2011 ▸). The details of the backing seat are described in Section S1 in the supporting information. A small ruby sphere was introduced in the sample space to estimate the sample pressure from the ruby fluorescent method (Piermarini *et al.*, 1975 ▸). The sample pressure for the diffraction measurements was determined as the average and the deviation between before and after the measurements.

**Figure 1:**
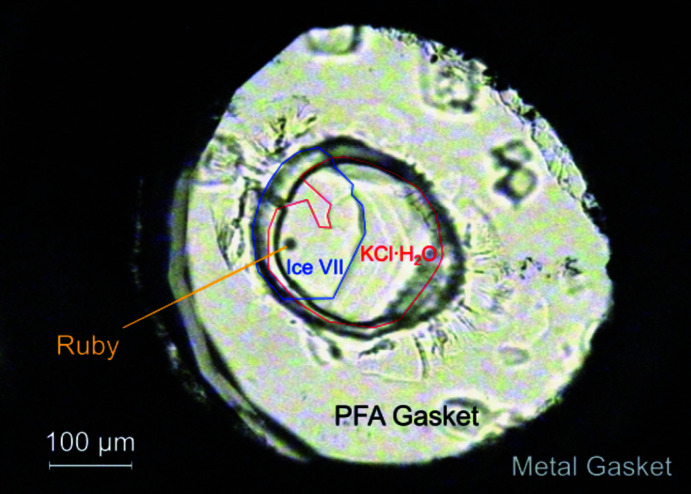
Photograph of single crystals of KCl·H_2_O with co-existing ice VII in a diamond anvil cell at 2.27 GPa and 295 K. The outlines of KCl·H_2_O and ice VII are highlighted by red and blue lines, respectively, as a guide for the eyes. A photograph without this eye guide is shown in Fig. S2 in the supporting information.

The sealed sample was compressed up to 2.4 GPa at 295 K and heated to ∼350 K. At these high-pressure and high-tem­per­ature conditions, single crystals of ice VII formed after cyclic compression and decompression. After the crystal growth of ice VII, the sample pressure decreased to ∼2.3 GPa at ∼320 K. The sample was compressed and heated again until the remaining solution started to freeze. Further compression and decompression were repeated to obtain single crystals of the KCl hydrate, co-existing with ice VII at 2.3 GPa and 295 K (Fig. 1 ▸).

Ideally, no co-existing crystals are preferred for measurements without interference from extra Bragg spots. However, water ices inevitably crystallize before the formation of KCl hydrate. We initially tested a KCl-saturated solution without KCl crystals as a starting material, but this resulted in its co-existence with ice VI. Ice VI has an orthorhombic structure with lattice parameters *a* ∼ 6.2 Å and *c* ∼ 5.7 Å, and their Bragg spots were harmful for indexing and intensity extraction of the Bragg peaks of the hydrate. We then decided to introduce additional crystalline KCl in a saturated solution to suppress the crystallization of water ice. The solubility of KCl increases up to a certain concentration upon compression. In the experiments, the measurement conditions were tuned to establish two requirements: (i) the co-existence with ice VII stable above 2 GPa rather than ice VI and (ii) the complete dissolution of the added KCl crystals into the solution before the formation of the hydrate. Ice VII has a highly symmetric structure with small lattice parameters of *a* ∼ 3.3 Å and exhibits a smaller number of Bragg spots than ice VI. Furthermore, the increase of KCl concentration is advantageous for the formation of a larger fraction of KCl hydrate in the sample space. In our preliminary experiments, the remaining crystalline KCl hardly transformed into the hydrate even co-existing with water ice.

The DAC containing the single-crystalline specimens was placed on an X-ray diffractometer (Rigaku, Synergy custom). The sample was irradiated with X-rays (Mo *K*α, λ = 0.7107 Å) from a micro-focused X-ray generator (Rigaku, MicroMax-007) and diffraction was detected with a hybrid photon counting X-ray detector (Rigaku, HyPix-6000HE). Experimental details and results are summarized in Table 1 ▸. The collected diffraction patterns were indexed and the diffraction intensities were extracted using *CrysAlis PRO* (Agilent, 2014 ▸). The diffraction intensities were corrected for attenuation by the diamond anvils using a self-made *ad hoc* program. Details of this program are described in Section S3 in the supporting information. In this correction procedure, unreasonable diffraction peaks out of the opening angle of the DAC were eliminated from the geometric calculations. Diffraction intensities less than 3σ were also eliminated to exclude dif­fractions which were accidentally blocked by the metal parts of the DAC or strongly attenuated by the metal gasket.

The initial structure of the hydrate was determined by direct methods using *SIR2018* (Burla *et al.*, 2015 ▸). The crystal structure without H atoms was refined using *SHELXL2018* (Sheldrick, 2015 ▸) within *WinGX* (Farrugia, 2012 ▸), without any parameter restrictions. The crystal structure models are described using *VESTA* (Momma & Izumi, 2011 ▸).

### DFT calculations

The crystal structure of the hydrate with H atoms was derived by a computational approach because of the difficulty in determining H-atom positions from *in situ* X-ray diffraction experiments, particularly under pressure. The structure was optimized by DFT calculations with the plane wave pseudopotential method (Hohenberg & Kohn, 1964 ▸; Kohn & Sham, 1965 ▸) using *Quantum ESPRESSO* (Giannozzi *et al.*, 2009 ▸, 2017 ▸). We used the generalized gradient approximation, GGA–PBE (Perdew *et al.*, 1996 ▸), with an energy cut-off of 150 Ry and 4 × 4 × 3 k-points. The structure model derived from the X-ray diffraction was used as the initial structure. The H atoms were located between the O and Cl atoms with *P*2_1_/*n* symmetry and an O-D distance of ∼1 Å. The structure was relaxed using the BFGS algorithm in a fixed cell determined from the X-ray diffraction measurements at 2.23 (4) GPa and 295 K. We also tested some initial structures with different alignments of the H atoms, but all of them converged into the same structure after the structure optimization. Robustness was examined using similar structure optimization with dispersion corrections DFT-D3 (Grimme *et al.*, 2010 ▸, 2011 ▸) and XDM (Becke & Johnson, 2005 ▸, 2007 ▸; Otero-De-La-Roza & Johnson, 2012 ▸). The XDM damping function parameters were set to their established literature values of *a*_1_ = 0.3275 and *a*_2_ = 2.7673 Å (Roza & DiLabio, 2017 ▸; Otero-De-La-Roza & Johnson, 2020 ▸).

## Results and discussion

### Crystal structure of KCl·H_2_O

**Figure 2:**
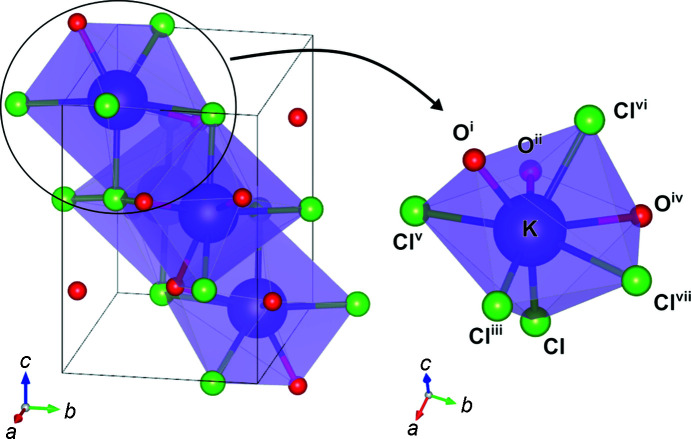
The crystal structure of KCl·H_2_O for a unit cell and extracted KCl_5_O_3_ undecahedron. H atoms which connect to O atoms are not described because they were not determined from the X-ray diffraction analysis. [Symmetry codes: (i) −*x* + 1, −*y* + 1, −*z* + 2; (ii) *x*, *y* − 1, *z*; (iii) *x* + 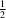 , −*y* + 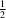 , *z* + 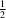 ; (iv) −*x* + 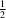 , *y* − 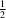 , −*z* + 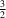 ; (v) −*x* + 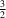 , *y* − 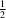 , −*z* + 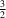 ; (vi) *x* − 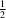 , −*y* + 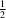 , *z* + 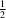 ; (vii) −*x* + 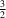 , *y* + 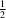 , −*z* + 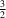 .]

The new potassium chloride hydrate has a structure with monoclinic symmetry (space group *P*2_1_/*n*; Fig. 2 ▸), determined from the systematic absences of diffractions: *h* + *l* ≠ 2*n* (*n* is an integer) for *h*0*l* and *k* ≠ 2*n* for 0*k*0. The nonstandard cell setting of *P*2_1_/*n* was selected to avoid extraordinarily large β ∼ 140° in the *P*2_1_/*c* unit cell. This nonstandard setting also has the advantage of avoiding interference with the error estimation of structure parameters due to the oblique setting (Feast *et al.*, 2009 ▸). This crystal lattice is distinct from those of anhydrous KCl and pure water ice. Considering the multiplicity of four for the general positions in *P*2_1_/*n*, the formula unit, *Z*, of this hydrate can be deduced to be 4. Its unit-cell volume of 293.7 (4) Å^3^ corresponds to the increase in molar volume by 24 Å^3^ with respect to the B2 phase at this pressure (Dewaele *et al.*, 2012 ▸). Considering a characteristic molecular volume for bound water in other inorganic hydrates (*e.g.* 26.17 Å^3^/H_2_O in MgSO_4_ hydrates at ambient pressure; Fortes *et al.*, 2012 ▸), we find that *n* = 1 and the crystal is, therefore, a monohydrate. Each K atom is surrounded by eight atoms: three O atoms and five Cl atoms (Fig. 2 ▸ and Table 2 ▸). On the other hand, each Cl atom is connected to five K atoms. Considering the sum of the covalent length for potassium and oxygen (∼2.7 Å), each O atom is considered to be shared by two K atoms like NaCl·2H_2_O (Klewe & Pedersen, 1974 ▸; Bode *et al.*, 2015 ▸), while the other K—O distance of 3.174 (7) Å would not be considered coordination, but accidental ap­proach by compression. In many known hydrates, such as magnesium salt hydrates (Hennings *et al.*, 2013 ▸), the cation atoms are six-coordinated by anions or water molecules, forming octahedra at ambient pressure regardless of the hydration numbers. Larger coordination numbers of cations can be seen in hydrates with large cations, such as calcium (Agron & Busing, 2002 ▸; Hennings *et al.*, 2014 ▸). Compared to octahedra, more highly coordinated polyhedra have lower symmetry but still tend to be somewhat symmetric to reduce the interatomic repulsions. KCl·H_2_O contains various types of interactions, *i.e.* ionic bonds, coordination, covalent bonds and hydro­gen bonds. Such inhomogeneity is considered to enhance the distortion of the KCl_5_O_3_ undecahedron, especially under high-pressure conditions.

### Structural relation with anhydrous KCl

Considering the small hydration number of KCl·H_2_O, its structure would be recognized as being mainly composed of cation–anion interactions like anhydrous salts rather than hydro­gen bonds in hydrates with fully hydrated cations, such as MgSO_4_·11H_2_O, MgCl_2_·10H_2_O and MgCl_2_·6H_2_O (Fortes *et al.*, 2008 ▸; Komatsu *et al.*, 2015 ▸; Yamashita *et al.*, 2019 ▸). In anhydrous KCl, K—Cl distances can be estimated to be ∼3.04 (B1 phase) and ∼3.18 Å (B2 phase) at ∼2.2 GPa and 298 K from their respective equations of state (Dewaele *et al.*, 2012 ▸). Two of the five K—Cl distances in KCl·H_2_O are close to 3.1 Å, while the other three are close to or longer than 3.2 Å (Table 2 ▸). The coordination of K—Cl in KCl·H_2_O can be explained as the intermediate state between the B1 phase, *i.e.* the ambient pressure phase, and the B2 phase, *i.e.* the high-pressure phase, of anhydrous KCl.

**Figure 3:**
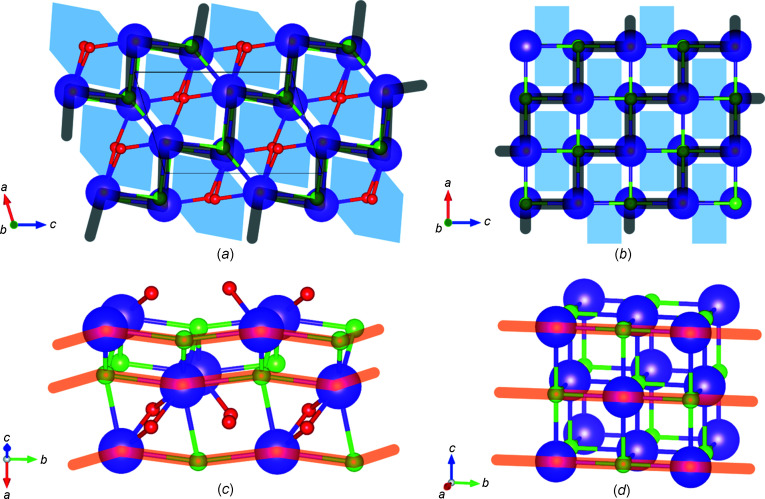
Structural comparison between KCl·H_2_O and the B1 phase of KCl. (*a*) KCl·H_2_O viewed along the *b* axis. (*b*) KCl viewed along the *b* axis. Bird views for (*c*) KCl·H_2_O and (*d*) KCl. Purple, green and red balls represent K, Cl and O atoms, respectively. H atoms of water molecules are not shown for clarity. Black lines indicate K–Cl chains with K—Cl distances shorter than 3.2 Å almost perpendicular to the *b* axis. Red lines show K–Cl chains along the *b* axis. The blue shaded areas represent a space elongating along the *b* axis of KCl·H_2_O, where water molecules are located.

In KCl·H_2_O, the K and Cl atoms with the shorter distances align in zigzag chains along *a* + *c* (Fig. 3 ▸). These zigzag chains have angles close to 90° for both K—Cl—K and Cl—K—Cl. Moreover, KCl·H_2_O also has almost straight K–Cl chains along the *b* axis [Fig. 3 ▸(*c*)]. In these K—Cl components, K and Cl atoms are directly connected along the three orthogonal directions. Such K—Cl alignments in the zigzag plane resemble the f.c.c. structure of anhydrous KCl (B1).

From the similarity of K—Cl alignments, the structure of KCl·H_2_O can be interpreted as a complex structure consisting of part of the B1 phase of the anhydrous salt and additional water molecules. In the view projected on the *ac* plane, water molecules are located between the chains. The zigzag K–Cl planes are displaced to make space for the intercalating water molecules, resulting in long K—Cl distances along almost perpendicular directions to the zigzag planes. Moreover, the water molecules are slightly displaced along the *b* axis located between K–Cl layers perpendicular to the *b* axis, resulting in long K—Cl distances along the *b* axis comparable to anhydrous KCl (B2).

### Computational structure evaluation of KCl·H_2_O

**Figure 4:**
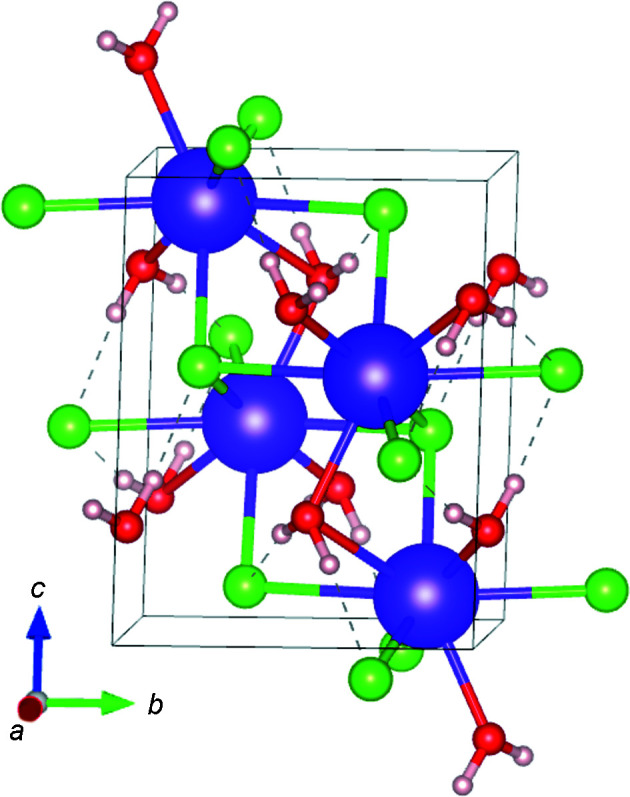
(*a*) The crystal structure of KCl·H_2_O optimized by DFT calculations using the PBE functional (Perdew *et al.*, 1996 ▸) with a fixed cell to be experimentally derived. Purple, green, red and pink balls represent K, Cl, O and H atoms, respectively

The crystal structure of KCl·H_2_O was also examined by DFT calculations (Fig. 4 ▸). The optimized atomic positions were in good agreement with the experimental results (Table S1 in the supporting information). The interatomic distances for K—Cl and K—O pairs (Table S2) indicate that the dispersion corrections did not act significantly to improve the reproducibility of geometries for the structure optimization with a fixed-cell constraint. Each H atom of the water molecules directly orients to the neighbouring Cl atom, with distances of 2.15 and 2.18 Å, which do not differ by more than 0.02 Å with or without the dispersion corrections (Table S2). These H⋯Cl distances are shorter than that of the known alkali halide hydrate, *i.e.* ∼2.4 Å in NaCl·2H_2_O (Bode *et al.*, 2015 ▸). MgCl_2_·6H_2_O has similar H⋯Cl distances and transforms into the high-pressure phase at 0.9 GPa (Yamashita *et al.*, 2019 ▸). During this transformation, the structure reduces its symmetry accompanied by a slight shortening of one of the two equivalent H⋯Cl distances to 2.1 Å, while the other equivalent distance elongates to 3.1 Å. Considering the elongation of some K—Cl distances, the short H⋯Cl distances in KCl·H_2_O would be the result of energy compensation for stress releases at different parts of the crystal structure including some repulsions and steric hindrances at specific parts.

## Concluding remarks

Potassium chloride monohydrate, KCl·H_2_O, was discovered by crystallization directly from a KCl solution under high pressure. *In situ* single-crystal diffraction revealed that its crystal structure comprises K—Cl alignments similar to that of the B1 phase of KCl. The zigzag K–Cl layers in the hydrate are separated by water molecules. The water intercalations elongate some K—Cl distances, resulting in the intermediate coordination structures of potassium and chlorine regarded as a mixture of structural components of the B1 and B2 phases of KCl, also supported by the obtained pressure of 2.23 (4) GPa close to the B1→B2 phase transition pressure of KCl. Such a structural relation of salt hydrates with an anhydrous salt would be applied to other cases, such as NaCl·2H_2_O, in which Na atoms form six-coordinated octahedra (Klewe & Pedersen, 1974 ▸; Bode *et al.*, 2015 ▸).

We finally note the possibility of other salt hydrates under high pressure. Despite the simplicity of the components, the number of known hydrates and their behaviours under high pressure are still limited for specific cases, such as MgSO_4_ and MgCl_2_ hydrates. As described in this and previous studies, multicomponent systems can form unique phases under high pressure, distinct from ambient-pressure phases in structure (Wang *et al.*, 2018 ▸; Yamashita *et al.*, 2019 ▸) or composition (Komatsu *et al.*, 2015 ▸; Fortes *et al.*, 2017*b* ▸). Tem­per­ature is also an important factor to determine the formation of hydrates. Low-tem­per­ature conditions are favoured for hydrates with higher hydration numbers at ambient pressure, as seen in the cases of MgCl_2_ hydrates (Hennings *et al.*, 2013 ▸). However, the formation of hydrates can be restricted kinetically, especially for the transition starting from a mechanical mixture of crystalline salt and water ice because the hydrate formation needs diffusion of chemical species. In nature, such transitions can proceed over geological timescales, but their experimental investigations are sometimes unachievable. Further investigations for high-pressure and low-tem­per­ature regions would need tricky approaches, such as crystallization from amorphous saline solution (Komatsu *et al.*, 2015 ▸).

## Related literature

The following reference is cited in the supporting information: Arndt *et al.* (2006 ▸).

## Supplementary Material

Crystal structure: contains datablock(s) global, I. DOI: 10.1107/S2053229622011135/ef3038sup1.cif

Structure factors: contains datablock(s) I. DOI: 10.1107/S2053229622011135/ef3038Isup2.hkl

Additional text, figures and tables. DOI: 10.1107/S2053229622011135/ef3038sup3.pdf

CCDC reference: 2220961

## Figures and Tables

**Table 1:** Crystallographic parameters and experimental details for the single-crystal X-ray diffraction experiments on KCl monohydrate

Chemical formula	KCl·H_2_O
*M*_r_	92.57
Pressure (GPa)	2.23 (4)
Tem­per­ature (K)	295
Crystal system, space group	Monoclinic, *P*2_1_/*n*
*a* (Å)	5.687 (7)
*b* (Å)	6.3969 (8)
*c* (Å)	8.447 (3)
β (°)	107.08 (8)
*V* (Å^3^)	293.7 (4)
*Z*	4
Calculated density (Mg m^−3^)	2.048
Specimen shape, size (µm)	Platelet, 220 × 180 × 90
	
Data collection	
Radiation type	Mo *Kα*, micro-focused
Data collection method	Rigaku, Hypix-6000HE
Exposure (s/frame)	120
Orientations	ω scans for 80 frames by 0.5°
μ (mm^−1^)	2.399
Range of *h*, *k*, *l*	−3 ≤ *h* ≤ 3 −7 ≤ *k* ≤ 7 −8 ≤ *l* ≤ 7
Number of measured, used and unique reflections	1507, 892, 147
	
Refinement	
Rejection criteria	*I* > 3σ
*R*_int_, *R*_σ_	0.1350, 0.0462
*R*_1_	0.0610
w*R*_2_	0.1984
*S*	1.797
Number of refined parameters, restraints	13, 0
Min/Max residual (e Å^−3^)	−0.34/0.56

Computer programs: *CrysAlis PRO* (Agilent, 2014 ▸) and *SHELXL2018* (Sheldrick, 2015 ▸).

**Table 2:** Selected bond lengths (Å) for KCl·H_2_O from X-ray diffraction experiments

K1—O1^i^	2.736 (6)	K1—O1^iv^	3.174 (7)
K1—O1^ii^	2.833 (8)	K1—Cl1^v^	3.1874 (15)
K1—Cl1	3.063 (5)	K1—Cl1^vi^	3.208 (6)
K1—Cl1^iii^	3.116 (6)	K1—Cl1^vii^	3.2637 (16)

Symmetry codes: (i) −*x* + 1, −*y* + 1, −*z* + 2; (ii) *x*, *y* − 1, *z*; (iii) *x* + 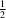 , −*y* + 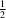 , *z* + 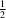 ; (iv) −*x* + 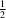 , *y* − 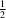 , −*z* + 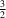 ; (v) −*x* + 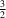 , *y* − 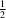 , −*z* + 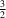 ; (vi) *x* − 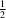 , −*y* + 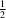 , *z* + 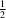 ; (vii) −*x* + 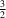 , *y* + 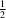 , −*z* + 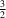 .
