# Crystal structure of potassium chloride monohydrate: water intercalation into the B1 structure of KCl under high pressure. Corrigendum

**DOI:** 10.1107/S2053229623000669

**Published:** 2023-01-30

**Authors:** Keishiro Yamashita, Kazuki Komatsu, Hiroyuki Kagi

**Affiliations:** a Geochemical Research Center, Graduate School of Science, The University of Tokyo, Hongo 7-3-1, Bunkyo-ku, Tokyo 113-0033, Japan; b Institute of Physical Chemistry, University of Innsbruck, Innrain 52c, Innsbruck, 6020, Austria

**Keywords:** salt hydrate, high pressure, intercalation, crystal structure, potassium chloride

## Abstract

In the paper by Yamashita *et al.* [ *Acta Cryst.* (2022), C**78**, 749–754], an incorrect phrase is updated.

## Abstract 2

Corrigendum to *Acta Cryst.* (2022), C**78**, 749–754.

In the first paragraph of the *Introduction* in the paper by Yamashita *et al.* (2022 ▸), the sentence ‘The low hydrate-forming capability of KCl can be ascribed to the energetic disadvantages of hydration, as seen in its **negative enthalpy of dissolution**’ is corrected to ‘The low hydrate-forming capability of KCl can be ascribed to the energetic disadvantages of hydration, as seen in its **endothermic dissolution**’; the updated phrase is highlighted in bold.

## References

[bb1] Yamashita, K., Komatsu, K. & Kagi, H. (2022). *Acta Cryst.* C**78**, 749–754. 10.1107/S2053229622011135 PMC9720884 36468558

