# Supplementary material for: Crystal structure of potassium chloride monohydrate: water intercalation into the B1 structure of KCl under high pressure
Source: Acta Crystallogr C Struct Chem. 2022 Dec 1;78(Pt 12):749–54. doi: 10.1107/S2053229622011135 (PMC9720884; doi:10.1107/S2053229622011135)
Supplement: Supplementary file 3 [file c-78-00749-sup3.pdf]

Acta Cryst C
STRUCTURAL
CHEMISTRY

Volume 78 (2022)

Supporting information for article:

Crystal structure of potassium chloride monohydrate: water intercalation into the B1 structure of KCl under high pressure

Keishiro Yamashita, Kazuki Komatsu and Hiroyuki Kagi

S1. ‘Clover seat’ – a design of backing seat for single-crystal X-ray diffraction

We newly designed a backing seat for diamond anvils, especially optimized for single-crystal X-ray diffraction to maximise the accessible opening angles for incoming and outgoing X-ray beam. The anvil and seat design are similar to the so-called Boehler-Almax type (Boehler & De Hantsetters, 2004), characterized by conical support. Then, our backing seat could be considered as a modified Boehler-Almax type. Here, we call our design ‘clover seat’ for its trefoil shape as described below. The concept of the design is similar to that we previously reported (Komatsu *et al.*, 2011), so that a part of the backing seat does not support the anvil, namely X-ray can travel through the unsupported part. The backing seat in the previous design has two opposed supported parts and also two unsupported parts (see Figure 1 in Komatsu *et al.*, 2011). The previous design successfully worked in terms of the wide accessibility to reciprocal space, but there was a problem with the achievable pressure; it was limited up to 5 GPa. We consider that the reason why the diamond broke at such low pressure could be the two-fold symmetry of the backing seat. Thus, we modified the backing seat to have three-fold symmetry as shown in Figure S1. The supported and unsupported part makes the opening angle up to ±51.5° and ±65° from the compression axis and open 50° and 70° each in a horizontal plane, respectively (Figure S1). This seat could have the world-most widely opened design, and we believe it will provide opportunities to study a large variety of crystals as well as salt hydrates in this study.

The backing seat is made of tungsten carbide, and the diamond anvil is manufactured to a conical shape, which is the same as Boehler-Almax type anvils. We confirmed that this set-up of the backing seat and anvils (culet diameter: ϕ0.6 mm) achieved at least up to 10 GPa without any visible damage. No further compression was tested.

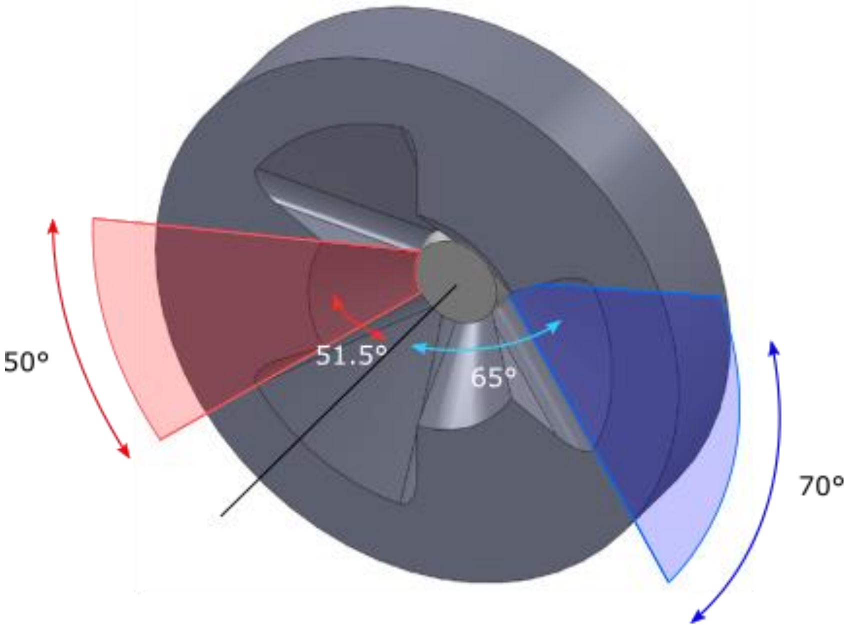

Figure S1 Schematic image of the 'clover seat' used in the single-crystal X-ray diffraction experiment. The shaded area in red or blue represents the supported or unsupported part respectively. Blackline is corresponding to the compression axis of the cell.

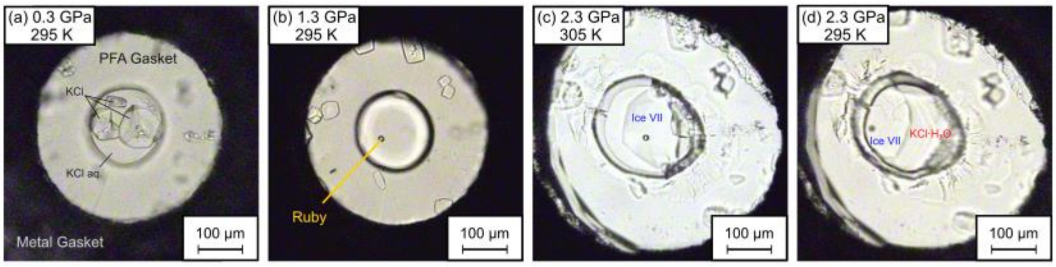

Figure S2 Photographs of sample space for growing crystals in a diamond anvil cell. (a) KCl solution with crystalline KCl. (b) KCl solution. (c) single-crystalline ice VII. (d) single-crystalline $KCl·H_2O$ with coexisting ice VII.

S2. Attenuation correction for diamond anvils

In the X-ray diffraction experiments, X-rays are inevitably attenuated by the diamond anvils on the way. These events occur for the incident and diffracted beams. The attenuation factor (A) can be calculated by the Beer-Lambert law ($A = e^{-\mu l}$) with the path length (l) and the attenuation coefficient of diamond (μ = 0.203 mm^{-1}: Arndt *et al.*, 2006). The path lengths depend on the directions of the X-ray with respect to the diamond anvils (Figure S3). In the self-made program, the corrected diffraction intensity (I_{corr}) is derived from each observed intensity (I_{obs}), unit vectors along the incident ($\boldsymbol{v}_{inc}$) and diffracted ($\boldsymbol{v}_{dif}$) X-rays described by the orthonormal basis for the reciprocal space of the specimen crystal, orientation matrix of the crystal ($\boldsymbol{U}$), and thicknesses of the diamond anvils (t_{inc} and t_{dif}) as

$$I_{corr} = I_{obs} \exp\left[\mu\left(\frac{t_{inc}}{|\boldsymbol{u}\cdot\boldsymbol{U}^{-1}\boldsymbol{v}_{inc}|} + \frac{t_{dif}}{|\boldsymbol{u}\cdot\boldsymbol{U}^{-1}\boldsymbol{v}_{dif}|}\right)\right],$$

where, $\boldsymbol{u}$ is a unit vector along the axial direction of the diamond anvils with thicknesses of t_{inc} and t_{dif} for the incident and diffracted X-rays, respectively. Diffraction peaks whose angles from the axial direction of the anvils exceeds the opening angle are eliminated as unobservable peaks.

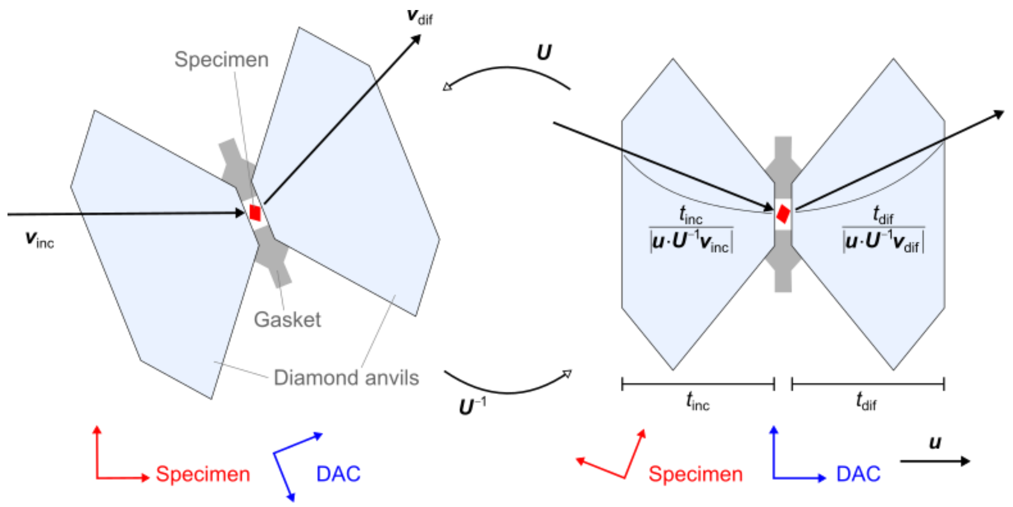

Figure S3 Schematic images for calculating the path length in the self-made program. The unit vectors for incident ($\boldsymbol{v}_{inc}$) and diffracted ($\boldsymbol{v}_{inc}$) beams are defined for the orthonormal basis of the reciprocal space of the specimen crystal. These vectors are projected to the coordinate defined by the orthonormal basis for the diamond anvil cell (DAC). Directional cosines of the incident and diffracted X-ray beams can be derived as the dot product of unit vectors along the X-rays and the axial direction of the diamond anvils.

S3. Optimised structure from DFT calculations

Table S1 Structure parameters of KCl·H_2O optimised by DFT calculation using the PBE-GGA functionals (Perdew *et al.*, 1996) with the cell constraint of a = 5.687 Å, b = 6.3969 Å, c = 8.447 Å, and β = 107.08°.

	x	Δx^*	y	Δy^*	z	Δz^*
K1	0.6899	-0.0003	0.1916	-0.0038	0.9314	-0.0013
Cl1	0.7472	0.0003	0.1984	-0.0030	0.5815	-0.0017
O1	0.2510	0.0074	0.9897	-0.0022	0.7621	-0.0041
H1	0.2802	-	0.9230	-	0.6641	0.2802
H2	0.0999	-	0.0729	-	0.7166	0.0999

*Difference from experimentally derived atomic coordinates.

Table S2 Interatomic distances of KCl·H_2O derived from the DFT structure optimisations with the cell constraint of a = 5.687 Å, b = 6.3969 Å, c = 8.447 Å, and β = 107.08°.

	PBE	PBE+XDM	PBE+D3	Exp.
K1—O1^{i}	2.766	2.794	2.774	2.736 (6)
K1—O1^{ii}	2.801	2.825	2.803	2.833 (8)
K1—Cl1	3.067	3.043	3.069	3.063 (5)
K1—Cl1^{iii}	3.128	3.108	3.121	3.116 (6)
K1—O1^{iv}	3.192	3.189	3.205	3.174 (7)
K1—Cl1^{v}	3.1802	3.2029	3.1804	3.1874 (15)
K1—Cl1^{vi}	3.212	3.230	3.216	3.208 (6)
K1—Cl1^{vii}	3.2673	3.2280	3.2692	3.2637 (16)
O1—H1	0.987	0.987	0.987	-
O1—H2	0.988	0.987	0.987	-
H1—Cl^{viii}	2.178	2.176	2.176	-
H2—Cl^{ix}	2.151	2.160	2.144	-

Symmetry code(s): (i) −*x*+1, −*y*+1, −*z*+2; (ii) *x*, *y*−1, *z*; (iii) *x*+1/2, −*y*+1/2, *z*+1/2; (iv) −*x*+1/2, *y*−1/2, −*z*+3/2; (v) −*x*+3/2, *y*−1/2, −*z*+3/2; (vi) *x*−1/2, −*y*+1/2, *z*+1/2; (vii) −*x*+3/2, *y*+1/2, −*z*+3/2; (viii) −*x*+1, −*y*+1, −*z*+1; (ix) *x*−1, *y*+1, *z*+1.

Selected geometric parameters (Å, º)

K1—O1^{i}	2.736 (6)	K1—Cl1^{vi}	3.208 (6)
K1—O1^{ii}	2.833 (8)	K1—Cl1^{vii}	3.2637 (16)
K1—Cl1	3.063 (5)	K1—O1^{viii}	3.395 (6)
K1—Cl1^{iii}	3.116 (6)	K1—K1^{ix}	3.697 (6)
K1—O1^{iv}	3.174 (7)	K1—K1^{x}	4.196 (6)
K1—Cl1^{v}	3.1874 (15)	K1—K1^{xi}	4.399 (4)
O1^{i}—K1—O1^{ii}	96.82 (13)	Cl1^{v}—K1—Cl1^{vii}	165.1 (3)
O1^{i}—K1—Cl1	151.3 (2)	Cl1^{vi}—K1—Cl1^{vii}	85.45 (14)
O1^{ii}—K1—Cl1	81.96 (15)	O1^{i}—K1—O1^{viii}	62.12 (7)
O1^{i}—K1—Cl1^{iii}	82.4 (2)	O1^{ii}—K1—O1^{viii}	122.1 (3)
O1^{ii}—K1—Cl1^{iii}	162.78 (19)	Cl1—K1—O1^{viii}	141.57 (14)
Cl1—K1—Cl1^{iii}	90.49 (17)	Cl1^{iii}—K1—O1^{viii}	72.79 (17)
O1^{i}—K1—O1^{iv}	128.8 (4)	O1^{iv}—K1—O1^{viii}	87.32 (12)
O1^{ii}—K1—O1^{iv}	64.38 (9)	Cl1^{v}—K1—O1^{viii}	129.44 (12)
Cl1—K1—O1^{iv}	76.58 (18)	Cl1^{vi}—K1—O1^{viii}	55.88 (16)
Cl1^{iii}—K1—O1^{iv}	128.90 (19)	Cl1^{vii}—K1—O1^{viii}	57.70 (16)
O1^{i}—K1—Cl1^{v}	67.57 (16)	K1—Cl1—K1^{xi}	90.79 (17)
O1^{ii}—K1—Cl1^{v}	67.60 (18)	K1—Cl1—K1^{vii}	95.62 (10)
Cl1—K1—Cl1^{v}	85.81 (9)	K1^{xi}—Cl1—K1^{vii}	83.45 (10)
Cl1^{iii}—K1—Cl1^{v}	96.55 (10)	K1—Cl1—K1^{xii}	134.59 (10)
O1^{iv}—K1—Cl1^{v}	130.52 (14)	K1^{xi}—Cl1—K1^{xii}	128.12 (11)
O1^{i}—K1—Cl1^{vi}	68.4 (2)	K1^{vii}—Cl1—K1^{xii}	70.63 (13)
O1^{ii}—K1—Cl1^{vi}	66.23 (18)	K1—Cl1—K1^{v}	94.08 (9)
Cl1—K1—Cl1^{vi}	134.18 (10)	K1^{xi}—Cl1—K1^{v}	107.69 (10)
Cl1^{iii}—K1—Cl1^{vi}	128.12 (11)	K1^{vii}—Cl1—K1^{v}	165.1 (3)
O1^{iv}—K1—Cl1^{vi}	60.50 (19)	K1^{xii}—Cl1—K1^{v}	94.55 (14)
Cl1^{v}—K1—Cl1^{vi}	109.37 (13)	K1^{i}—O1—K1^{xiii}	83.18 (13)
O1^{i}—K1—Cl1^{vii}	119.07 (18)	K1^{i}—O1—K1^{xiv}	95.9 (3)
O1^{ii}—K1—Cl1^{vii}	121.89 (17)	K1^{xiii}—O1—K1^{xiv}	169.0 (3)
Cl1—K1—Cl1^{vii}	84.49 (9)	K1^{i}—O1—K1^{xv}	169.7 (3)

$Cl1^{iii}$—K1—$Cl1^{vii}$	72.31 (10)	$K1^{xiii}$—O1—$K1^{xv}$	89.4 (2)
$O1^{iv}$—K1—$Cl1^{vii}$	57.52 (15)	$K1^{xiv}$—O1—$K1^{xv}$	92.68 (12)

Symmetry code(s): (i) −*x*+1, −*y*+1, −*z*+2; (ii) *x*, *y*−1, *z*; (iii) *x*+1/2, −*y*+1/2, *z*+1/2; (iv) −*x*+1/2, *y*−1/2, −*z*+3/2; (v) −*x*+3/2, *y*−1/2, −*z*+3/2; (vi) *x*−1/2, −*y*+1/2, *z*+1/2; (vii) −*x*+3/2, *y*+1/2, −*z*+3/2; (viii) *x*+1/2, −*y*+3/2, *z*+1/2; (ix) −*x*+1, −*y*, −*z*+2; (x) −*x*+2, −*y*, −*z*+2; (xi) *x*−1/2, −*y*+1/2, *z*−1/2; (xii) *x*+1/2, −*y*+1/2, *z*−1/2; (xiii) *x*, *y*+1, *z*; (xiv) −*x*+1/2, *y*+1/2, −*z*+3/2; (xv) *x*−1/2, −*y*+3/2, *z*−1/2.
